# Supplementary material for: Applying a system dynamics approach for decision-making in software testing projects
Source: PLoS One. 2025 May 16;20(5):e0323765. doi: 10.1371/journal.pone.0323765 (PMC12084065; doi:10.1371/journal.pone.0323765)
Supplement: Attachment — Submitted filename: PLOS_The Detailed Response to Reviewers.docx [file pone.0323765.s006.docx]

**The Detailed Response to Reviewers (and Editors)**

**Manuscript ID: PONE-D-24-47186**

**Title: Applying a System Dynamics Approach for Decision-Making in Software Testing Projects**

Dear AE,

Thanks for the insightful and constructive comments made by the reviewers. The manuscript has been substantially revised according to the reviewers’ comments, and the following pages summarize our explanations and the revision works. Note that all the revisions are marked with red texts in the manuscript.

Below is a summary of the key points raised by the reviewers and our responses:

**Reviewer #1:**

1. Highlighting Contributions: We have revised the last two paragraphs of the introduction to explicitly outline the three key contributions of our study: (1) integrating dynamic interactions among testing, debugging, and resource allocation; (2) enhancing practical relevance by incorporating real-world factors; and (3) accounting for the learning curve of personnel.

2. Improving Readability: We have divided longer sentences throughout the manuscript, especially in the abstract and key sections, to improve clarity and flow.

3. Model Advantages: We have expanded the discussion on how our system dynamics model addresses the limitations of traditional models, especially in scenarios involving multiple testing teams and imperfect debugging.

4. Model Verification: We explained why metrics like predictive power (PP) and predictive risk ratio (PRR) are not suitable for our study due to the characteristics of time-series software testing data. Additionally, we corrected editorial errors in Table 3.

5. Strengthening Conclusions: We enhanced the conclusion by discussing both the short-term and long-term implications of our research for project management, particularly in relation to resource allocation and data-driven decision-making.

**Reviewer #2:**

1. Practical Deployment Scenarios: Although our primary focus is on methodological development, we have included a detailed hypothetical example in Section 4 to demonstrate the practical application of our model in a real-world project setting.

2. Imperfect Debugging: We modified the model to accommodate imperfect debugging by integrating stochastic elements into the time-dependent function A(t), thereby enhancing the simulation's alignment with real-world practices.

3. Agile Methodologies: We recognize the significance of Agile practices; however, we clarified that incorporating them into our existing framework necessitates additional research. We intend to investigate this further in future studies.

4. Benchmarking: We clarified our selection of NHPP-based models for benchmarking and explained why FeSAD frameworks are not directly applicable to our study.

5. Statistical Techniques: We have included a discussion on the potential application of Bayesian inference and Monte Carlo simulations in future research to enhance parameter estimation under conditions of uncertain data.

6. Scalability: We have included a discussion on how our model can be adapted for large-scale distributed systems, including the potential integration of cloud-based testing tools and distributed computing.

**Reviewer #1:**

**Questions:**

1. The introduction establishes the importance of software quality, but it needs to highlight the contribution of your study.

**REPLY:**

Thank you for your suggestion. We have rewritten the last two paragraphs of the introduction to highlight the contributions of our study. The content of the revised paragraphs is as follows:

The study aims to integrate real-world scenarios into the system dynamics model, thereby providing a more realistic and practical perspective. This includes considering factors such as team capabilities, resource limitations, and market pressures, all of which can significantly impact the reliability of software and the testing processes. In summary, the study introduces an innovative framework for decision-making in software testing projects by applying a system dynamics approach, offering three key contributions to the field. First, the proposed methodology presents a comprehensive scientific management framework that integrates dynamic interactions among software testing, debugging processes, and resource allocation. Second, the model enhances practical relevance by incorporating a broader array of real-world factors, such as evolving team dynamics, imperfect debugging scenarios, and changing project constraints. Third, the study also accounts for the learning curve of testing and debugging personnel, allowing for a more accurate representation of improvements in software reliability throughout the testing process.

We hope that the revised texts can address your concerns. If there are any deficiencies, we can make further improvements.

Please see page 3 for the revision.

2. Consider breaking up longer sentences for improved readability. For instance, the sentence starting with “However, the challenging balance...” could be split for clarity.

**REPLY:**

Thank you for your valuable feedback and constructive suggestions. I have carefully reviewed your comment regarding the readability of longer sentences, particularly the example you provided starting with “However, the challenging balance...”. In response, I have made extensive revisions throughout the manuscript, including breaking up longer sentences into shorter, more concise ones to enhance clarity and flow. I hope these changes address your concern and improve the overall readability of the paper.

I appreciate your time and effort in helping me refine this work. Please let me know if there are any additional adjustments needed.

Please see the abstract and pages 2, 4, 7, and 18 for the revisions.

3. Figure 1 is the good addition. You mention that traditional models are complex and impractical. Expanding on how your model specifically addresses these shortcomings would strengthen your argument and provide a clearer rationale for your approach.

**REPLY:**

Thank you for your questions. In traditional models, all testing teams are treated as a single entity, and the parameters of the software reliability growth model are estimated based on this collective unit. However, in practice, testing teams often undergo changes and reorganization. In such cases, the model parameters previously estimated for the unified team are no longer applicable and must be re-estimated, as the values of these parameters have shifted. Furthermore, when considering multiple testing teams and the possibility of imperfect testing introducing new software defects, it becomes challenging to estimate testing efficiency using past mathematical inference methods. This is because deriving a closed-form mathematical model under these conditions is highly complex. Please refer to Figure A, which illustrates that each testing team’s testing efficiency can be described using a mathematical model. However, when considering the scenario of imperfect debugging across all teams, the combined mathematical models do not yield a closed-form expression to fully characterize the entire situation. To address this limitation, we utilize a system dynamics model, which allows for a comprehensive and dynamic representation of the entire testing process.

We have addressed these issues in the revised manuscript, and we hope that the modifications provide readers with a clearer understanding of the subject.

Figure A. Causal Loop Diagram Model with Multiple Teams for Software Testing Process

Please refer to page 10 for the revisions.

4. Paper working on Decision-Making in Software Testing Projects, so some similar work may be considered in the introduction section and also in total expected cost analysis part.

(i) Pradhan, V., Dhar, J., & Kumar, A. (2022). Software reliability models and multi-attribute utility function based strategic decision for release time optimization. In Predictive Analytics in System Reliability (pp. 175-190).

Cham: Springer International Publishing.

(ii) Pradhan, V., Kumar, A., & Dhar, J. (2023). Emerging trends and future directions in software reliability growth modeling. Engineering reliability and risk assessment, 131-144.

**REPLY:**

We sincerely thank the reviewer for recommending the relevant literature. These references have effectively addressed the gaps identified in our Introduction, enriching the depth and comprehensiveness of our literature review. We have incorporated the suggested references into the manuscript and updated the Reference list accordingly.

Please refer to pages 2 and 21 for the revisions.

5. In Subsection 2.3 Model Verification and Comparison, more comparison methods are required like predictive power (PP) and predictive risk ratio (PRR). See the comparison of R-square of proposed model and Pham and Zhang [5] are same for all the datasets.

**REPLY:**

Thank you for your questions. To evaluate metrics such as predictive power (PP) and predictive risk ratio (PRR), it is essential to divide the original data into two distinct sets: the model construction data (training data) and the testing data. This division is crucial for ensuring the validity and reliability of the predictive metrics.

However, in publicly available software testing datasets, the data consists of time-series records of testing activities. Extracting any segment of this time-series data for testing purposes would create a gap in the training data, leading to significant bias in the model parameters. Given this characteristic of software testing data, metrics such as predictive power (PP) and predictive risk ratio (PRR) are not suitable for our research problem. This is why these two metrics are rarely used to evaluate predictive performance in related studies. Instead, most research in this field relies on measures of fitting ability, such as mean squared error (MSE) and R-squared, to assess model performance. For this reason, our study adopts the evaluation metrics commonly used in the majority of related research.

Besides, thank you for bringing the issue regarding Table 3 to our attention. In the previous version, there were some editorial errors in Table 3, which have now been corrected. Additionally, the identical R-square values observed across different models are due to the precision being limited to three decimal places.

Please refer to Table 3 on page 9 for the revisions.

6. Your conclusion touches on the impact of your research on decision-making. Strengthen this section by discussing potential implications for project management and strategies in both the short and long term.

**REPLY:**

Thank you for your constructive feedback on strengthening the discussion of managerial implications. We have revised the Conclusions section to explicitly address short-term and long-term project management strategies, including:

(1) Short-term strategies: Project managers can utilize the model to optimize the allocation of testing resources, prioritize high-impact debugging tasks, and adjust timelines based on real-time reliability predictions. For example, the cost estimation framework (Objective 2) facilitates rapid “what-if” analyses for budget adjustments during sprint cycles.

(2) Long-term strategies: Organizations can adopt the system dynamics approach to institutionalize data-driven decision-making. This may include aligning team training programs with the learning curves identified in the model (Objective 1) and establishing iterative feedback loops between testing outcomes and process improvements.

Please refer to page 19 for the revisions.

**Reviewer #2:**

**General Comment:** After a detailed analysis of the uploaded document, here are six critical recommendations to address the identified shortcomings, along with relevant research papers for further exploration.

**Questions:**

1. The study presents theoretical models for software reliability but lacks practical deployment scenarios. Include real-world case studies demonstrating the model’s application in diverse industries, such as financial systems or healthcare software.

**REPLY:**

Thank you for your valuable feedback regarding the inclusion of practical deployment scenarios in our study. We appreciate your suggestion to incorporate real-world case studies from diverse industries, such as financial systems or healthcare software, to demonstrate the model’s applicability.

However, we would like to clarify that the primary focus of this research is to propose a methodological framework that addresses the limitations of traditional models in estimating software reliability and testing costs, particularly in scenarios involving multiple testing teams. This methodology is designed to provide software testing managers with a flexible and adaptive tool for effective resource allocation to achieve project goals.

To illustrate the practical application of our approach, we included a detailed example in Section 4 (Application and Numerical Analysis). In this section, we modeled a scenario where a software development company is tasked with developing an Enterprise Resource Planning Information System (ERPIS) project. The example demonstrates how our methodology can be used to formulate a software testing plan, optimize resource allocation, and predict reliability and costs in a real-world project setting. While this example is hypothetical, it is grounded in realistic assumptions and reflects the challenges faced by software testing managers in practice.

We acknowledge that including actual deployment data from real-world projects, such as financial or healthcare systems, could further strengthen the persuasiveness of our findings. However, as our research primarily emphasizes the development and application of the methodology, we have not sought to publicly disclose proprietary data from specific companies. We believe that the hypothetical example provided in Section 4 effectively showcases the practicality and relevance of our approach without compromising the confidentiality of real-world data.

We hope that the example in Section 4, along with the methodological contributions of our study, sufficiently demonstrates the value and applicability of our framework. We kindly request to retain the current content, as it aligns with the research’s focus on advancing a flexible and adaptive methodology for software reliability and testing cost estimation.

Thank you again for your thoughtful comments. Please let us know if further clarifications or adjustments would enhance the manuscript.

2. While the paper explores mathematical models, it assumes “perfect debugging,” which is often unrealistic. Discuss how factors like imperfect debugging and evolving software environments impact the model’s effectiveness. Incorporate contemporary approaches such as dynamic fault modeling or machine learning-driven predictions.

**REPLY:**

Thank you for your insightful questions. While the foundational mathematical model was initially derived under the assumption of perfect debugging, our subsequent derivations allow for the transformation of the number of initial errors ($A$) into a time-dependent function ($A\left( t \right)$). This enables the conversion of the original perfect debugging Software Reliability Growth Model (SRGM) into an imperfect debugging SRGM. We apologize for not clearly explaining this in the previous version of the manuscript. In this revised version, we have added and modified relevant discussions to ensure clarity for readers. Furthermore, in our system dynamics model, we have incorporated stochastic elements into the function $A\left( t \right)$. Although $A\left( t \right)$ increases over time, it follows a probability distribution, making the simulation scenarios more aligned with real-world practices.

Regarding your mention of machine learning-driven predictions, we acknowledge their effectiveness in predicting software reliability. However, since our methodology is based on system dynamics, integrating machine learning approaches into our model is not straightforward. In future research, we aim to explore ways to combine system dynamics with machine learning, which could potentially enhance the predictive accuracy of the model.

Please refer to pages 5, 6, 7, 11, and 12 for the revisions.

3. Most modern software development adopts Agile methodologies, but the study does not address iterative testing scenarios. Suggest adaptations of the proposed models for continuous integration and deployment pipelines.

**REPLY:**

Thank you for your insightful comment regarding the application of our proposed models to Agile methodologies and iterative testing scenarios.

However, we would like to clarify that the primary focus of this study is to develop a methodological framework for estimating software reliability and testing costs in scenarios involving multiple testing teams. While Agile methodologies and multi-version software development are highly relevant and important topics, integrating these aspects into our current research framework presents significant theoretical and practical challenges. Our methodology is designed to address specific gaps in traditional reliability models, and extending it to accommodate Agile practices would require substantial additional research and validation.

That said, we fully acknowledge the importance of exploring these issues in future work. Your suggestion aligns with our long-term research objectives, and we envision adapting the proposed models to better fit iterative development environments. This could involve incorporating dynamic feedback loops, version-specific reliability tracking, and real-time data integration to enhance support for Agile workflows.

Thank you once again for your constructive feedback.

4. The manuscript evaluates the proposed model but does not adequately benchmark it against state-of-the-art reliability growth models (SRGMs). Include comparative results with models like NHPP and FeSAD frameworks to validate the model’s superiority.

**REPLY:**

Thank you for your valuable feedback regarding the benchmarking of our proposed model against state-of-the-art reliability growth models (SRGMs). We appreciate your suggestion to include comparative results with models such as NHPP and FeSAD frameworks to validate the model's performance.

Regarding FeSAD, we are uncertain whether this refers to Federated Learning and Secure Data (a framework often associated with distributed machine learning and data security). If that is the case, we would like to clarify that FeSAD frameworks, while highly relevant in the context of federated learning and secure data processing, do not directly align with the focus of our study. Our research centers on traditional SRGMs, particularly those based on Non-Homogeneous Poisson Process (NHPP) models, which are widely utilized in software reliability prediction.

As for benchmarking, we acknowledge the importance of comparing our model with existing SRGMs. However, due to the numerous NHPP-based SRGMs, we focused on selecting representative and widely recognized models from recent literature to ensure a meaningful and manageable comparison.

Thank you once again for your constructive feedback. Please let us know if any further clarifications or adjustments would improve the manuscript.

5. The reliance on maximum likelihood estimation (MLE) and least squares estimation (LSE) is limiting. Introduce additional statistical techniques like Bayesian inference or Monte Carlo simulations to improve parameter estimation accuracy under uncertain data conditions.

**REPLY:**

Thank you for your insightful comment and valuable suggestion regarding the use of additional statistical techniques, such as Bayesian inference and Monte Carlo simulations, to enhance parameter estimation accuracy under uncertain data conditions.

In the current study, our foundational model is based on nonlinear regression, and the parameter estimation methods employed are MLE and LSE, which are widely used and well-established in the field of software reliability modeling. While these methods are effective under conditions where sufficient historical data is available, we acknowledge that they may face challenges in scenarios with limited or uncertain data.

In response to your suggestion, we have revised the Conclusions and Future Directions section to explicitly highlight the potential of Bayesian inference and Monte Carlo simulations as promising avenues for future work. We aim to explore these methods in subsequent studies to enhance the model’s robustness and applicability in data-constrained environments.

Please refer to page 20 for the revisions.

6. The proposed approach should explicitly discuss scalability for large-scale distributed systems. Suggest integrating cloud-based testing tools or distributed computing to handle extensive datasets and real-time analytics.

**REPLY:**

Thank you for your valuable suggestion regarding the scalability of the proposed system dynamics model for large-scale distributed systems. We agree that integrating cloud-based testing tools and distributed computing frameworks is essential to handle extensive datasets and performing real-time analytics. In response to your feedback, we have added the related discussions in the manuscript to outline how the proposed model can be adapted for large-scale applications. These enhancements will enable the system dynamics model to address the increasing complexity and scale of the challenges.

Please refer to pages 13 for the revisions.

7. Update your literature and study the following papers:

(i)https://scholar.google.com/citations?view_op=view_citation&hl=en&user=Ztcq8g8AAAAJ&citation_for_view=Ztcq8g8AAAAJ:hqOjcs7Dif8C

(ii)https://scholar.google.com/citations?view_op=view_citation&hl=en&user=Ztcq8g8AAAAJ&citation_for_view=Ztcq8g8AAAAJ:IWHjjKOFINEC

(iii)https://scholar.google.com/citations?view_op=view_citation&hl=en&user=Ztcq8g8AAAAJ&citation_for_view=Ztcq8g8AAAAJ:W7OEmFMy1HYC

**REPLY:**

We sincerely thank the reviewer for recommending the relevant literature. These references have effectively addressed the gaps identified in our Introduction, enriching the depth and comprehensiveness of our literature review. We have incorporated the suggested references into the manuscript and updated the Reference list accordingly.

Please refer to pages 2 and 22 for the revisions.
